# The clinical characteristics of Chinese Takayasu’s arteritis patients: a retrospective study of 411 patients over 24 years

**DOI:** 10.1186/s13075-017-1307-z

**Published:** 2017-05-25

**Authors:** Jing Li, Fei Sun, Zhe Chen, Yunjiao Yang, Jiuliang Zhao, Mengtao Li, Xinping Tian, Xiaofeng Zeng

**Affiliations:** Department of Rheumatology and Clinical Immunology, Peking Union Medical College Hospital, Peking Union Medical College and Chinese Academy of Medical Sciences, Key Laboratory of Rheumatology and Clinical Immunology, Ministry of Education, Beijing, 100032 China

**Keywords:** Takayasu’s arteritis, Clinical characteristics, Angiography manifestation, Carotid artery, Aneurysm

## Abstract

**Background:**

We aimed to investigate the clinical characteristics of 411 Chinese Takayasu’s arteritis (TAK) patients using a retrospective analysis.

**Methods:**

We retrospectively reviewed 810 medical charts of patients with a diagnosis of TAK who were admitted to Peking Union Medical College Hospital from 1990 to 2014. 411 patients with a complete dataset were finally included in the analysis. The demographic data, clinical features, angiographic patterns, and TAK-related surgical procedures were collected and analyzed.

**Results:**

The median age at disease onset was 23 (18, 30) years old, with a median disease duration of 21 (6, 60) months; 325 (79.1%) were female. The angiographic involvement pattern was type I in 91 (22.1%) patients, type IIa in 16 (3.9%) patients, type IIb in 16 (3.9%) patients, type III in 12 (2.9%) patients, type IV in 26 (6.3%) patients, and type V in 250 (60.8%) patients. Subclavian arteries (79.8%) were the most commonly involved, followed by carotid arteries (79.1%). The occurrence rate (4.1%) of aortic aneurysm in this study was low; 119 operations and interventions were performed. The most common cause of death in this study was heart failure.

**Conclusion:**

Subclavian arteries, carotid arteries, and type V were the most frequently involved arteries and angiography pattern in this Chinese TAK study. The difference in angiographic features may lead to differences in clinical manifestations. Surgical operation and interventions should be performed at different stages of the disease course.

## Background

Takayasu’s arteritis (TAK) is an uncommon systemic inflammatory vasculitis with granulomatous inflammation in the adventitia and media of the aorta and its major branches [1]. The estimated annual incidence of TAK is 0.4–1.0 per million people in Germany [2], 2.0 per million in southeast Norway [3], and 3.4 per million in the northwestern part of Turkey [4]. In the UK primary care cohort, the prevalence is only 4.7 per million [5], but it is more common in southeast Asia, India, Japan, and Mexico [4]. The prevalence of TAK was estimated to be 40 per million in Japan [6]. In an autopsy series from Japan, the prevalence was reported to be as high as 1 in 3000 cases [7]. In the northwestern part of Turkey, the prevalence (33 per million) of TAK is higher than that of the western population (4.7–8.0 per million) [5, 8–10], but is similar to east Asia [4].

TAK mainly involves the aorta and its major branches. Fibrosis develops gradually as the disease progresses, followed by stenosis or occlusion of the vessels, which in turn results in organ ischemia [11]. Occasionally, the destruction of the elastica and muscularis may result in artery dilation or aneurysm [11]. The prevalence of aneurysm in Southern African patients [12], Thai patients [13], and Chinese patients [14, 15] seems to be very different. The angiographic patterns also vary in different ethnic groups [16]. In this study, we investigated the angiographic involvement patterns, clinical features, and the outcome of Chinese TAK patients, and also compared our data with the different ethnic groups reported in the literature.

## Methods

### Patients

We retrospectively reviewed 810 medical charts of patients with a diagnosis of TAK who were admitted to Peking Union Medical College Hospital (PUMCH), a Chinese national referral center, from 1990 to 2014. Charts of the same patients for multiple admissions were considered as a single case. Thus, finally, 411 patients with a complete dataset were included in this study, and the angiography manifestations of each patient with the most extended blood vessels involved were analyzed as some patients had more than one angiography evaluation performed during follow-up.

### Methods

A case search was performed electronically via the information systems of our hospital using The International Classification of Diseases Tenth Revision (ICD-10) code for TAK (M31.4). The diagnosis was confirmed through a chart review by two senior rheumatologists according to the 1990 American College of Rheumatology (ACR) criteria for TAK [17]. The information for these patients during their admission(s) in our center was collected. A database file was built under the guidance of one senior rheumatologist according to the information needs of this study. The data were entered into the database by four junior rheumatologists using Epidata (Version 3.1), and data were exported to SPSS software for further analysis. The input data in the database were mutual checked by all four rheumatologists to ensure their accuracy and completeness.

The demographic data, clinical presentations, physical examination findings, laboratory tests, angiographic involvement patterns, interventions, and surgical procedures were collected and analyzed. Renal dysfunction was defined as estimated glomerular filtration rate (eGFR) <90 ml/min/1.73 m^2^. Heart failure was defined as ejection fraction ≦40% by echocardiography or typical clinical symptoms (i.e., circulatory congestion, exertional dyspnea, orthopnea, etc.) with rales heard on a physical examination, with or without pleural effusion seen on chest radiography [18]. The angiographic involvement pattern was based on the 1994 Numano et al. criteria [19], and the description records of angiography were analyzed. Each patient had blood vessel examinations either by catheter angiography or computed tomography angiography (CTA) at least once to assess the extent of blood vessel involvement. If patients had symptoms that suggested possible intra-cranial, pulmonary, or coronary vessel involvement, then CTA or magnetic resonance angiography (MRA) for intra-cranial, pulmonary, or coronary arteries were performed. This is the general protocol for all inpatients with TAK. However, this may underestimate the prevalence of the special vessel involvement if the vessel change is not severe enough to cause clinical symptoms.

We searched PubMed with the keywords “Takayasu’s arteritis” and “cohort study” to obtain angiographic manifestations of other ethnic groups. The inclusion criteria for patients in these studies were all based on the 1990 ACR criteria for TAK, and patient numbers in studies over 50 were included in this study. Comparisons were made between this study and the studies we obtained from the literature.

### Statistical analysis

Because some of the data were not distributed in a normal pattern, we described the numerical variables as median (Q1, Q3), and the categorical variables as number (percentage). Comparisons between different populations were made using Chi-square tests for categorical data. Fisher’s exact tests were conducted when the expected frequencies were less than 5. A two-sided *P* value less than 0.05 was considered to be statistically significant. Analysis was performed with the SPSS software (version 19.0, IBM SPSS statistics, Armonk, New York, USA).

## Results

### Demographic data and clinical manifestations

The male to female ratio of this study was 1:3.8 (325 female, 86 male), with the median age of disease onset being 23 years old and the median duration of disease from onset 21 months. The clinical features and angiographic classification are presented in Table 1. Type V (60.8%) was the most common angiographic involvement pattern. Fever (31.1%) was the most common constitutional symptom, followed by malaise (29.7%) and weight loss (20.0%). Claudication, vessel bruits, and other symptoms/signs due to deficiency of arterial supply were common. Heart lesions and renal involvement due to hypertension were observed in this group of patients. The median levels of erythrocyte sedimentation rate (ESR), C-reactive protein (CRP), and high-sensitive CRP (hs-CRP) were all elevated (Table 1).

**Table 1:** Demographic data and clinical features of 411 Chinese TAK patients

	Patient number
Gender	
Female	325 (79.1%)
Male	86 (20.9%)
Age at onset (years)	23.0 (18.0, 30.0)^a^
Duration of disease at first admission (months)	21.0 (6.0, 60.0)^a^
Constitutional findings	
Fever	128 (31.1%)
Malaise	122 (29.7%)
Weight loss	82 (20.0%)
Symptoms	
Arthralgia	37 (9.0%)
Skin rash	32 (7.8%)
Vascular findings	
Bruit of carotid arteries	257 (62.5%)
Hypertension	209 (50.9%)
Asymmetric pulsation	158 (24.6%)
Claudication	
Upper limbs	60 (14.6%)
Lower limbs	57 (13.9%)
Gangrene	2 (0.5%)
Cardiac findings	
Valvar lesions	201 (48.9%)
Left ventricle hypertrophy on ECG	58 (14.4%)
Heart failure	61 (14.8%)
Pericardial effusion	30 (7.3%)
Myocardial lesions	26 (6.3%)
Angina	14 (3.4%)
Myocardial infarction	3 (0.7%)
Renal abnormalities	
Nephrotic-range proteinuria	13 (3.2%)
Renal dysfunction	47 (11.4%)
Neurological symptoms	
Headache	125 (30.4%)
Ischemic stroke	22 (5.4%)
Syncope	49 (11.9%)
Ophthalmoscope findings	
Hypertensive retinopathy	31/165^b^ (18.8%)
TAK-related retinopathy	23/165^b^ (13.9%)
Ischemic changes	11/165^b^ (6.7%)
Vasculitis presentations	11/165^b^ (6.7%)
Others	3/165^b^ (1.8%)
Angiographic classification	
Type I	91 (22.1%)
Type IIa	16 (3.9%)
Type IIb	16 (3.9%)
Type III	12 (2.9%)
Type IV	26 (6.3%)
Type V	250 (60.8%)
Laboratory findings	
Level of ESR (mm/h)	26.0 (11.0, 65.0)^a^
Level of CRP (mg/L)	12.8 (3.4, 44.8)^a^
Level of hs-CRP (mg/L)	8.8 (2.0, 12.5)^a^
Level of WBC (10^9^/L)	8.2 (6.6, 11.0)^a^
Level of Hgb (g/L)	121 (108, 135)^a^

^a^Median (Q1, Q3)

^b^Actually detected

*ECG* electrocardiogram, *ESR* erythrocyte sedimentation rate, *Hgb* hemoglobin, *hs-CRP* high-sensitive C-reactive protein, *TAK* Takayasu’s arteritis, *WBC* white blood cell

### Features of vessel involvement and distributions

The detailed distribution of vessel involvement is shown in Table 2. Among them, subclavian arteries (79.8%) and carotid arteries (79.1%) were the most commonly involved arteries, almost at the same frequency.

**Table 2:** The manifestations of vessels involved in 411 Chinese Takayasu’s arteritis patients

Arteries	Any arterial lesion	Stenosis	Occlusion	Dilatation	Aneurysm
Subclavian artery	328 (79.8%)	232 (56.4%)	130 (31.6%)	11 (2.7%)	5 (1.2%)
Left	283 (68.9%)	185 (45.0%)	110 (26.8%)	4 (1.0%)	3 (0.7%)
Right	223 (54.2%)	145 (35.3%)	58 (14.1%)	8 (1.9%)	3 (0.7%)
Carotid artery	325 (79.1%)	241 (58.6%)	102 (24.8%)	16 (3.9%)	3 (0.7%)
Left common	291 (70.8%)	175 (42.6%)	82 (20.0%)	6 (1.5%)	0
Right common	266 (64.7%)	156 (38.0%)	53 (12.9%)	9 (2.2%)	3 (0.7%)
Left internal	85 (20.7%)	56 (13.6%)	19 (4.6%)	5 (1.2%)	0
Right internal	94 (22.9%)	61 (14.8%)	15 (3.6%)	7 (1.7%)	0
Left external	59 (14.4%)	36 (8.8%)	14 (3.4%)	2 (0.5%)	0
Right external	55 (13.4%)	38 (9.2%)	9 (2.2%)	1 (0.2%)	0
Renal artery	201 (48.9%)	182 (44.3%)	43 (10.5%)	6 (1.5%)	2 (0.5%)
Left	157 (38.2%)	143 (34.8%)	24 (5.8%)	5 (1.2%)	1 (0.2%)
Right	149 (36.3%)	133 (32.4%)	24 (5.8%)	1 (0.2%)	1 (0.2%)
Abdominal aorta	158 (38.4%)	134 (32.6%)	12 (2.9%)	16 (3.9%)	10 (2.4%)
Vertebral artery	118 (28.7%)	82 (20.0%)	46 (11.2%)	10 (2.4%)	1 (0.2%)
Left	94 (22.9%)	62 (15.1%)	34 (8.3%)	6 (1.5%)	1 (0.2%)
Right	81 (19.7%)	55 (13.4%)	23 (5.6%)	10 (2.4%)	0
Mesenteric artery	116 (29.7%)	90 (21.9%)	33 (8.0%)	1 (0.2%)	0
Thoracic aorta	86 (17.5%)	72 (17.5%)	0	14 (3.4%)	3 (0.7%)
Innominate artery	81 (19.7%)	61 (14.8%)	16 (3.9%)	8 (1.9%)	1 (0.2%)
Iliacofemoral artery	65 (15.8%)	43 (10.5%)	18 (4.4%)	0	4 (1.0%)
Left	53 (12.9%)	32 (7.8%)	15 (3.6%)	0	3 (0.7%)
Right	54 (13.1%)	36 (8.8%)	11 (2.7%)	0	2 (0.5%)
Ascending aorta	39 (9.5%)	4 (1.0%)	0	37 (9.0%)	3 (0.7%)
Aortic arch	32 (7.8%)	25 (6.1%)	0	7 (1.7%)	1 (0.2%)
Intracranial artery^a^	31 (7.5%)	29 (7.1%)	2 (0.5%)	0	0
Pulmonary artery^b^	31 (7.5%)	17 (4.1%)	14 (3.4%)	1 (0.2%)	0
Left	22 (5.4%)	12 (2.9%)	6 (1.5%)	1 (0.2%)	0
Right	27 (6.6%)	12(2.9%)	11 (2.7%)	1 (0.2%)	0
Coronary artery^c^	15 (3.6%)	14 (3.4%)	4 (1.0%)	0	0
Left	13 (3.2%)	10 (2.4%)	3 (0.7%)	0	0
Right	7 (1.7%)	7 (1.7%)	1 (0.2%)	0	0

^a^131 patients with imaging of intracranial artery

^b^45 patients with imaging of pulmonary arteries

^c^42 patients with imaging of coronary arteries

We found another six cohort studies from different geographic areas [12, 20–24] which also described the details of involved vessels. The occurrence rate of carotid artery and subclavian artery involvement was similarly high, but the occurrence rates of aortic involvement and aortic aneurysm were different (Table 3).

**Table 3:** Manifestation of involved vessels in Takayasu’s arteritis patients from different geographic areas

Arteries	This study (*n* = 411)	Mwipatayi et al. [12] (*n* = 272)	Lee et al. [22] (*n* = 204)		Schmidt et al. [21] (*n* = 126)		Vanoli et al. [24] (*n* = 104)		Arnaud et al. [23] (*n* = 82)		Freitas et al. [20] (*n* = 52)	
			Left	Right	Left	Right	Left	Right	Left	Right	Left	Right
Subclavian artery	328 (79.8%)	ND	67.1%	55.2%	66.3%	41.0%	65.6%	52.5%	68.3%	50.0%	60.6%	40.4%
Stenosis	232 (56.4%)	ND	26.0%	24.6%	43.3%	36.2%	42.6%	29.5%	ND		25.0%	26.9%
Occlusion	130 (31.6%)	ND	34.8%	14.3%	29.8%	4.8%	23.0%	23.0%	ND		25.0%	5.8%
Dilatation	11 (2.7%)	ND	0.5%	4.4%	ND	ND	0	0	ND		0	5.8%
Aneurysm	5 (1.2%)	ND			0	1.0%	1.6%	0	ND		1.9%	1.9%
Carotid artery	325 (79.1%)	83 (30.5%)	72.1%^f^	63.7%^f^	50.9%	41.7%	44.3%	36.1%	59.8%^f^	52.4%^f^	57.3%	40.4%
Stenosis/occlusion	260 (63.3%)	59 (21.7%)	ND	ND	ND	ND	ND	ND	ND		ND	ND
Stenosis	241 (58.6%)	ND	33.3%^f^	32.3%^f^	41.7%	37.0%	37.7%	23.0%	ND		30.8%	23.1%
Occlusion	102 (24.8%)	ND	21.1%^f^	9.8%^f^	10.2%	5.6%	4.9%	6.6%	ND		9.6%	9.6%
Dilatation	16 (3.9%)	ND	1.0%^f^	1.5%^f^	ND	ND	0	3.3%	ND		1.9%	0
Aneurysm	2 (0.5%)	17 (6.3%)			0.9%	0.9%	1.6%	3.3%	ND		5.8%	1.9%
Aneurysm and stenosis/occlusion	1 (0.2%)	7 (2.6%)	ND	ND	ND	ND	ND	ND	ND		ND	ND
Renal artery	201 (48.9%)	ND	32.2%	31.7%	18.7%	20.9%	34.4%	29.5%	14.6%	15.9%	34.6%	36.5%
Stenosis	182 (44.3%)	ND	25.2%	25.2%	16.5%	19.8%	31.2%	23.0%	ND		15.4%	25.0%
Occlusion	43 (10.5%)	ND	6.0%	4.5%	4.4%	2.2%	3.3%	6.6%	ND		9.6%	3.8%
Dilatation	6 (1.5%)	ND	0	0	ND	ND	0	0	ND		0	0
Aneurysm	2 (0.5%)	ND	0	0	0	0	0	0	ND		0	0
Abdominal aorta	158 (38.4%)	186 (68.4%)	63.2%		23.7%^c^	27.4%^d^	39.3%		43.9%		63.5%	
Stenosis/occlusion	140 (34.1%)	115 (42.3%)	ND		ND	ND	ND		ND		ND	
Stenosis	134 (32.6%)	ND	38.3%		20.4%^c^	25.3%^d^	29.5%		ND		26.9%	
Occlusion	12 (2.9%)	ND	4.0%		1.1%^c^	2.1%^d^	6.6%		ND		1.9%	
Aneurysm	6 (1.4%)	41 (15.1%)	6.0%		2.2%^c^	1.1%^d^	3.3%		ND		7.7%	
Dilatation	0	ND			ND	ND	ND		ND		11.5%	
Aneurysm and stenosis/occlusion	4 (1.0%)	30 (11.0%)	ND		ND	ND	ND		ND		ND	
Vertebral artery	118 (28.7%)	ND	ND		18.5%	13.0%	13.3%	11.7%	28.0%	15.9%	25.0%	11.5%
Stenosis	82 (20.0%)	ND	ND		15.7%	9.3%	8.3%	8.3%	ND		13.5%	5.8%
Occlusion	46 (11.2%)	ND	ND		2.8%	3.7%	5.0%	1.7%	ND		3.8%	5.8%
Dilatation	10 (2.4%)	ND	ND		ND	ND	0	1.7%	ND		1.9%	0
Aneurysm	1 (0.2%)	ND	ND		0	0	0	0	ND		0	0
Mesenteric artery	116 (29.7%)	101 (37.1%)	22.8%^a^	3.5%^b^	24.7%^a^	6.9%^b^	31.6%^a^	9.4%^b^	ND		28.8%^a^	3.8%^b^
Stenosis/occlusion	116 (29.7%)	92 (33.8%)	ND	ND	ND	ND	ND	ND	ND		ND	ND
Stenosis	90 (21.9%)	ND	14.4%^a^	0.5%^b^	18.0%^a^	2.3%^b^	24.6%^a^	5.7%^b^	ND		15.4%^a^	3.8%^b^
Occlusion	33 (8.0%)	ND	5.9%^a^	2.0%^b^	6.7%^a^	4.6%^b^	7.0%^a^	1.9%^b^	ND		11.5%^a^	0^b^
Dilatation	1 (0.2%)	ND	1.0%^a^	1.0%^b^	ND	ND	0^a^	1.9%^b^	ND		0^a^	0^b^
Aneurysm	0	9 (3.3%)			0^a^	0^b^	0^a^	0^b^	ND		0^a^	0^b^
Thoracic aorta	86 (17.5%)	158 (58.1%)	57.2%		19.1%		11.1%		40.2%		34.6%	
Stenosis/occlusion	72 (17.5%)	98 (36.0%)	ND		18.2%		ND		ND		ND	
Stenosis	72 (17.5%)	ND	22.9%		18.2%		7.4%		ND		9.6%	
Occlusion	0	ND	0		0		0		ND		0	
Dilatation	14 (3.4%)	ND	3.0%		ND		1.9%		ND		9.6%	
Aneurysm	2 (0.5%)	38 (14.0%)			0.9%		1.9%		ND		3.8%	
Aneurysm and stenosis/occlusion	1 (0.2%)	22 (8.1%)	ND		ND		ND		ND		ND	
Innominate artery	81 (19.7%)	43 (10.5%)	46.8%		25.5%		8.8%		28.0%		25.0%	
Stenosis/occlusion	74 (18.0%)	22 (8.1%)	ND		ND		ND		ND		ND	
Stenosis	61 (14.8%)	ND	16.3%		18.9%		8.8%		ND		11.5%	
Occlusion	16 (3.9%)	ND	3.0%		6.6%		0		ND		1.9%	
Dilatation	8 (1.9%)	ND	4.0%		ND		1.8%		ND		7.7%	
Aneurysm	1 (0.2%)	16 (5.9%)			0.9%		0		ND		1.9%	
Aneurysm and stenosis/occlusion	0	5 (1.8%)	ND		ND		ND		ND		ND	
Iliacofemoral artery	65 (15.8%)	74 (27.2%)	13.3%^g^	14.8%^g^	13.5%^g^	13.5%^g^	19.7%^g^	18.9%^g^	12.2%^g^	18.3%^g^	23.1%^g^	23.1%^g^
Stenosis	43 (10.5%)	ND	6.9%^g^	8.9%^g^	10.1%^g^	10.1%^g^	9.8%^g^	6.6%^g^	ND		11.5%^g^	9.6%^g^
Occlusion	18 (4.4%)	ND	4.9%^g^	3.4%^g^	4.5%^g^	2.2%^g^	8.2%^g^	9.8%^g^	ND		5.8%^g^	5.8%^g^
Dilatation	0	ND	0.5%^g^	0^g^	ND	ND	1.6%^g^	1.6%^g^	ND		0^g^	0^g^
Aneurysm	4 (1.0%)	ND			0^g^	2.2%^g^	0^g^	0^g^	ND		0^g^	0^g^
Ascending aorta	39 (9.5%)	65 (23.9%)	47.8%		9.1%		ND		ND		30.8%	
Stenosis	3 (0.7%)	15 (5.5%)	0		2.7%		ND		ND		1.9%	
Dilatation	37 (9.0%)	ND	25.4%		ND		ND		ND		19.2%	
Aneurysm	2 (0.5%)	43 (15.8%)			6.4%		ND		ND		3.8%	
Aneurysm and stenosis	1 (0.2%)	7 (2.6%)	ND		ND		ND		ND		ND	
Aortic arch	32 (7.8%)	90 (33.1%)	37.9%		4.5%		10.3%		37.8%		19.2%	
Stenosis	25 (6.1%)	45 (16.5%)	0.5%		2.7%		5.2%		ND		1.9%	
Dilatation	7 (1.7%)	ND	3.4%		ND		5.2%		ND		9.6%	
Aneurysm	1 (0.2%)	36 (13.2%)			1.8%		0		ND		1.9%	
Aneurysm and stenosis	0	9 (3.3%)	ND		ND		ND		ND		ND	
Pulmonary artery	31/45^e^ (68.9%)	24 (8.8%)	13.4%		6/18^e^ (33.3%)		ND		ND		ND	
Stenosis/occlusion	26/45^e^ (57.8%)	13 (4.8%)	ND		ND		ND		ND		ND	
Stenosis	17/45^e^ (37.8%)	ND	4.0%		6/18^e^ (33.3%)		ND		ND		ND	
Occlusion	14/45^e^ (31.1%)	ND	2.0%		2/18^e^ (11.1%)		ND		ND		ND	
Dilatation	1/45^e^ (2.2%)	ND	2.0%		ND		ND		ND		ND	
Aneurysm	0	6 (2.2%)			0		ND		ND		ND	
Aneurysm and stenosis/occlusion	0	5 (1.8%)	ND		ND		ND		ND		ND	
Coronary artery	15/42^e^ (35.7%)	ND	31/49^e^ (63.3%)		4/18^e^ (22.2%)		ND		ND		ND	
Stenosis	14/42^e^ (33.3%)	ND	26/49^e^ (53.1%)		4/18^e^ (22.2%)		ND		ND		ND	
Occlusion	4/42^e^ (9.5%)	ND	2/49^e^ (4.1%)		1/18^e^ (5.6%)		ND		ND		ND	
Dilatation	0	ND	3/49^e^ (6.1%)		ND		ND		ND		ND	
Aneurysm	0	ND			0		ND		ND		ND	

^a^Superior mesenteric artery

^b^Inferior mesenteric artery

^c^Suprarenal aorta

^d^Infrarenal aorta

^e^Actually detected

^f^Common carotid artery

^g^iliac artery

*ND* no data

Ten studies from 11 different ethnic groups were found [13, 15, 16, 25–28], which also described the angiographic involvement pattern based on the classification of Numano et al. [19]. In eight groups, type V was the most common pattern, as seen in the present study, but in the other three groups, type I was the most common pattern (Table 4).

**Table 4:** Angiographic classification of Takayasu’s arteritis patients from different populations

	China/this study	Turkey [16]	Korea [22]	US [21]	China [15]	Mexico [34]	Korea [25]	India [26]	Japan [26]	Thailand [13]
*n*	411	248	204^d^	126^e^	125	110	108	102	79	63
Disease durations (months)	21.0 (6.0, 60.0)^a^	34.2 (0, 240)^b^	ND	13.3 (6.1, 35.5)^a^	19 (0.5, 160)^b^	ND	13.9 ± 10.1 (1, 60)^b^	ND	24.3 ± 11.5^c^	ND
Type I (%)	22.1	32	11.1	20	40	19	36.1	6.9	24.1	0
Type IIa (%)	3.9	6.9	8.6	6	4.8	3	2.8	1	11.4	0
Type IIb (%)	3.9	3.2	14.1	7	1.6	4	4.6	5.9	10.1	11.1
Type III (%)	2.9	3.2	4.0	5	2.4	4	7.4	2.9	0	3.2
Type IV (%)	6.3	3.7	7.6	5	20.8	2	15.7	28.4	1.3	19
Type V (%)	60.8	51	54.5	57	30.4	69	33.3	54.9	53.2	66.7

^a^Median (Q1,Q3)

^b^Median (range)

^c^Years

^d^198 patients had undergone angiographic evaluation

^e^100 patients had undergone angiographic evaluation

*ND* no data

### TAK-related surgical procedures and interventions

In this study, 119 operations and interventions were performed at different stages of disease, including 54 (13.1%) bypass surgery, 25 (6.1%) percutaneous transluminal angiography (PTA) for renal artery stenosis or occlusion, 22 (5.4%) PTA for stenosis or occlusion in other arteries, 35 (8.5%) stent implantation for stenosis, and 11 (2.7%) other operations for TAK-related lesions, including five valve replacements, four nephrectomies, and two repairs of aortic aneurysm (Table 5).

**Table 5:** The surgical and interventional operations taken in 411 Chinese Takayasu’s patients

Category of operations/interventions	Patient numbers	%
Operations on cervical vessels	53	12.9
Bypass between aorta and carotid artery	21	5.1
Bypass between carotid and subclavian arteries	11	2.7
Bypass between aorta and subclavian arteries	6	1.5
Angioplasty and/or stenting in carotid arteries	6	1.5
Angioplasty and/or stenting in subclavian arteries	4	1.0
Angioplasty and/or stenting in innominate arteries	3	0.7
Bypass between innominate and carotid arteries	1	0.2
Bypass between innominate and subclavian arteries	1	0.2
Operations to reduce renal hypertension	33	8.0
Angioplasty and/or stenting in renal arteries	25	6.1
Bypass between aorta and renal arteries	4	1.0
Nephrectomy	4	1.0
Operations on aortic and iliacofemoral stenosis or occlusion	20	4.9
Angioplasty and/or stenting in aorta	12	2.9
Aorta bypass grafting	5	1.2
Bypass between aorta and bilateral iliac arteries	1	0.2
Bypass between aorta and bilateral femoral arteries	1	0.2
Angioplasty and/or stenting in common iliac arteries	1	0.2
Operations on valvar lesions and coronary artery	9	2.2
Cardiac valve replacement	5	1.2
Angioplasty and/or stenting in coronary arteries	3	0.7
Coronary artery bypass grafting	1	0.2
Repair on aortic aneurysm	2	0.5
Ascending aorta and aortic arch	1	0.2
Thoracic and abdominal aorta	1	0.2
Rebuilding circulation in upper limbs	1	0.2
Bypass between axillary arteries	1	0.2
Rebuilding circulation in mesenteric arteries	1	0.2
Mesenteric artery bypass	1	0.2
Summary		
Bypass operations	54	13.1
Percutaneous transluminal angiography (PTA)	47	11.4
PTA in renal arteries	25	6.1
Stenting	35	8.5
Other operations	11	2.7

### Mortality rate and causes of death

In this study, 12 patients deceased at a median age of 33.5 (19.8, 59.8) years old. The median survival time was 102.5 (46.0, 242.5) months. The direct causes of death were heart failure in five, bleeding in two, pulmonary infection in two, sudden cardiac death due to severe pulmonary hypertension in one, postoperative complication in one, and end-stage malignancy in one patient [29].

## Discussion

As a rare systemic large vessel vasculitis, most reported studies in the literature on TAK are small in sample size. In China, the prevalence of TAK is relative higher than many other geographic areas, such as Europe and North America. In this study, we retrospectively analyzed the data of 411 inpatients with complete angiographic description. According to our knowledge, this is the largest series of patients with TAK who were admitted to a rheumatology department thus far.

TAK can be divided into two phases, the acute phase and the chronic or stenotic phase, based on clinical presentations. The acute phase, which is characterized by nonspecific constitutional symptoms caused by acute inflammation, is not evident in the majority of TAK patients. Most patients have no evident acute phase; the initial symptoms are manifestations caused by organ ischemia due to vessel stenosis or occlusion. Constitutional presentations, which are mainly due to inflammation, are found in more than one-third of patients in this study. This is parallel to the laboratory findings, including elevated level of ESR and hs-CRP (Table 1).

Through the literature review, we found that involvement of carotid arteries and subclavian arteries were similarly high in the seven studies [12, 20–24], including the present one (Table 3). It is widely accepted that carotid arteries are the most commonly involved arteries in TAK patients. The symptoms and findings from physical examination of carotid arteries are usually obvious and lead to the consideration of the diagnosis of TAK. But the symptoms of subclavian arteries are insidious until severe ischemia develops, presenting as claudication of the upper limbs, imbalance of pulsation, or differences in bilateral blood pressure. The lesions of subclavian arteries are occasionally missed during screening of TAK-related arterial presentation by Doppler ultrasound because of the technical difficulties. Because of the high occurrence rate of subclavian arterial lesions in TAK, we suggest that careful screening of subclavian arterial lesions should be done, especially when patients present with symptoms or signs suggesting subclavian arterial ischemia.

In general, the major angiographic features of TAK are artery stenosis and occlusion, with relatively few dilatations and aneurysms. However, in the study by Mwipatayi and co-researchers [12], 305 aneurysms were found in 272 TAK patients. However, in Chinese cohort studies, the reported occurrence of aneurysm ranged from 3.4% to 4.8% [14, 15]. Even in different areas of Asia, the occurrence rate of aneurysms varied markedly. In a cohort study from Thailand [13], 38 aneurysms were found in 63 TAK patients. In the Chinese cohort reported by Cong et al. [15], 6 aneurysms were found in 125 TAK patients. In the present study, 33 (8.0%) aneurysms were found in 411 Chinese TAK patients (Table 2), which is similar to the study of Cong et al. but much lower than in the study of Mwipatayi et al. or the Thai study [12, 13]. This may suggest that genetic factors might play a role in the pathogenesis of TAK. The high prevalence of TAK in Asia may be related to environmental factors, such as the prevalence of tuberculosis infection [30], which was assumed to be associated with TAK pathogenesis and development [31], while no direct evidence was found in arterial lesions of TAK patients [32, 33].

We compared the angiographic classification pattern of our patients with nine groups of TAK patients from different geographic areas in eight reported studies [13, 15, 16, 22, 23, 25, 26, 34] (Table 4). The most common pattern in seven of the studies was type V, as in this study, while in the other two groups it was type I. This difference might result from the selection of patients at different stages of TAK development. Since TAK is a chronic inflammatory disease, patients are prone to have the type I pattern in the early stage of TAK, while type V may be seen in the late stage. Autopsy in Japanese TAK patients showed evidence that various stages of inflammation existed in the aortic walls, including infiltration of inflammatory cells, granulomatosis, and fibrosis [35]. The new active lesions were usually found adjacent to the fibrotic ones [35], which suggested consecutive inflammatory progression in the vessel wall in the involved arteritis. Therefore, a longer disease duration was associated with more extended lesions in TAK. As the disease duration of our patients was usually long, this may be the reason that type V was the most common pattern in this study.

In this study, bypass surgery (54/411, 13.1%) was the most common surgical intervention for TAK-related lesions, followed by PTA (25/411, 6.1%) to relieve refractory hypertension caused by severe renal artery stenosis (Table 3). This is consistent with the distribution pattern of involved vessels in this group of patients. Stenosis of carotid arteries, subclavian arteries, and renal arteries was found in 58.6%, 56.4%, and 44.3% of patients, respectively, which were the three most commonly involved arteries in this study (Table 2). Dizziness or upper limb intermittent claudication is the most prominent clinical manifestation of stenosis of cervical vessels, which may be the initial clinical presentation that drives patients to seek medical support. However, the leading causes for surgical operations were renovascular hypertension (48/155, 31.0%), hypertension due to aortic coarctation (32/155, 20.6%), and carotid arterial occlusion (26/155, 16.8%) in the report by Miyata et al. [36]. The difference in the occurrence rates of carotid stenosis lesion and aortic coarctation in different ethnic groups would lead to the difference in the rate of surgical intervention for TAK patients.

The most common cause of death in this study was heart failure, which was secondary to hypertension and aortic regurgitation [29]. In this study, which included TAK patients admitted to PUMCH over the past 24 years, the main direct cause of death was heart failure (4/12, 33.3%), followed by hemorrhagic shock (2/12, 16.7%) due to gastrointestinal tract bleeding, and septicemia secondary to pulmonary infection (2/12, 16.7%). Heart failure was secondary to hypertension and aortic regurgitation in most of the deceased patients. These suggest that controlling blood pressure to the normal range may be important in preventing the death of TAK patients. The hemorrhagic complications might be related to the use of low-dose aspirin (75 or 100 mg/day) in TAK patients; however, de Souza and co-researchers have proven that antiplatelet therapy was associated with a lower frequency of ischemic events in patients with TAK [37]. Thus they suggested that the use of antiplatelet therapy in TAK patients was more beneficial than harmful.

The major limitation of this study is that only inpatients were included. Therefore, the selection of the study patients was biased. The retrospective analysis and chart review placed the study at the risk of recall bias, which may compromise the power of the conclusion. There was also a lack of standardized protocol to evaluate the arterial lesions. Further prospective longitudinal study is needed.

## Conclusions

In conclusion, blood vessel involvement in Chinese TAK patients is different from other ethnic groups. Aortic aneurysm is less common in Chinese TAK patients, while the subclavian artery and carotid artery may be more commonly involved in Chinese TAK patients. In addition, when compared with other studies, type V is more common in Chinese TAK patients. The difference in angiographic features may lead to the difference in clinical manifestations, and also the difference in surgical interventions. The most common cause of death in Chinese TAK patients in this study is heart failure secondary to hypertension caused by renal artery involvement, which suggests that more attention should be paid to controlling blood pressure in the normal range.

## Abbreviations

CRP: C-reactive protein
CTA: Computed tomography angiography
ESR: Erythrocyte sedimentation rate
hs-CRP: High-sensitive C-reactive protein
MRA: Magnetic resonance angiography
PTA: Percutaneous transluminal angiography
TAK: Takayasu’s arteritis
